# Risk factors for open-angle glaucoma in Nigeria: results from the Nigeria National Blindness and Visual Impairment Survey

**DOI:** 10.1186/s12886-016-0264-7

**Published:** 2016-06-07

**Authors:** Fatima Kyari, Mohammed M. Abdull, Richard Wormald, Jennifer R. Evans, Winifred Nolan, Gudlavelleti V. S. Murthy, Clare E. Gilbert, On behalf of the Nigeria National Blindness and Visual Impairment Study Group, Abdullahi Imam, Adenike Abiose, Christian Ezelum, Gabriel Entekume, Hannah Faal, Mansur M. Rabiu, Olufunmilayo O. Bankole, Pak Sang Lee, Tafida Abubakar

**Affiliations:** International Centre for Eye Health (ICEH), Department of Clinical Research, London School of Hygiene & Tropical Medicine (LSHTM), Keppel Street, London, WC1E 7HT UK; Department of Ophthalmology, College of Health Sciences (CHS), University of Abuja, Abuja, Nigeria; Department of Ophthalmology, Abubakar Tafawa Balewa University Teaching Hospital, Bauchi, Nigeria; Moorfields Eye Hospital, London, UK; Indian Institute of Public Health, Public Health Foundation of India, Hyderabad, Telangana State India

**Keywords:** Open-angle glaucoma, Risk factors, Ethnicity, Nigeria

## Abstract

**Background:**

The glaucoma-specific blindness prevalence in Nigeria (0.7 %, 95 % CI 0.6–0.9 %) among those aged ≥40 years is one of the highest ever reported. This study determined the risk factors for open-angle glaucoma (OAG) in adults examined in the Nigeria National Blindness and Visual Impairment Survey.

**Methods:**

A nationally representative sample of 13,591 people aged ≥40 years in 305 clusters in Nigeria were examined (response rate 90.4 %) between January 2005 to June 2007. Everyone had logMAR visual acuity measurement, Frequency Doubling Technology (FDT) visual field testing, autorefraction, A-scan biometry and optic disc assessment. Full ocular examination (*n* = 6397), included Goldmann applanation tonometry. Values for defining glaucoma using International Society of Geographical and Epidemiological Ophthalmology criteria were derived from the study population. Disc images were graded by Moorfields Eye Hospital Reading Centre. Socio-demographic factors (age, gender, ethnicity, literacy and place of residence), ocular parameters (intraocular pressure [IOP], axial length and mean ocular perfusion pressure [MOPP]) and systemic parameters (blood pressure, blood glucose and body mass index [BMI]) were assessed for association with OAG.

**Results:**

Thirteen thousand eighty-one (96 %) of 13,591 participants had vertical cup:disc ratio measured in at least one eye. 682 eyes of 462 participants were classified as OAG, with 12,738 controls. In univariate analyses the following were associated with OAG: increasing age, male gender, Igbo and Yoruba ethnic groups, illiteracy, longer axial length, higher IOP, lower MOPP, greater severity of hypertension and low BMI (underweight). In multivariate analysis, increasing age (odds ratio [OR] 1.04, 95 % CI 1.03–1.05), higher IOP (OR 1.22, 95 % CI 1.18–1.25) and Igbo ethnicity (OR 1.73, 95 % CI 1.18–2.56) were independent risk factors for OAG.

**Conclusion:**

Case detection strategies for OAG should be improved for those aged ≥40 years and for ethnic groups most at risk as a public health intervention.

## Background

In 2013 it was estimated that there were 64.3 million people aged 40–80 years with glaucoma worldwide, projected to increase to 76.0 million by the year 2020 and 111.8 million in 2040 [1]. Open-angle glaucoma (OAG) is the most prevalent type of glaucoma in Africa [1–6] and a leading cause of blindness and visual impairment [2, 7]. The glaucoma-specific blindness prevalence in Nigeria (0.7 %, 95 % confidence interval [CI] 0.6–0.9 %) among those aged 40 years and above is one of the highest ever reported [8], and glaucoma is the second-leading cause of blindness after cataract [8]. The all glaucoma prevalence in Nigeria in this age-group was 5.02 % (95 % CI 4.60–5.47 %), with 86 % being OAG based on gonioscopy. An estimated 1.2 million adults in Nigeria had glaucoma in 2012 [9].

There are some similarities in the epidemiology of OAG in sub-Saharan African and Caribbean populations. An interesting aspect of the Barbadian history is that a significant portion of the population was derived from the Bight of Biafra (also known as Bight of Bonny) in southeastern Nigeria; and about 44 % of enslaved Africans taken to Barbados during the 18^th^ century were said to be mainly of Igbo origin [10]. Studies of risk factors for OAG in sub-Saharan Africa and African-derived black populations have reported that increasing age [3–6, 11–13] and higher intra-ocular pressures (IOP) [3, 4, 12, 14] are consistent and important risk factors. Although not always observed, men have a higher prevalence of glaucoma [4, 5, 12, 15]. A consistent finding is a higher prevalence of OAG in blacks compared to whites in populations where the two racial groups were studied [11, 13, 15]. The prevalence of glaucoma was higher in those with darker skin and of African birth [13], which suggest possible influence of environmental factors and inter-ethnic variation in the prevalence and risk of OAG within black populations, mediated by genetic factors. A higher prevalence of OAG in the urban population of Chennai compared to the rural population suggest a possible influence of lifestyle differences and non-communicable diseases such as hypertension and diabetes which are also more prevalent in the urban population [16]. Very few studies have explored other socio-demographic and systemic risk factors.

The Nigeria National Blindness and Visual Impairment Survey (hereafter referred to as the Nigeria Blindness Survey) is one of the largest population-based survey ever undertaken in Africa [17]. The present paper analysed data from the Nigeria Blindness Survey to explore risk factors for OAG among adults aged ≥40 years. Factors other than age and IOP were assessed. Identifying population groups most at risk, such as ethnic groups, will aid in planning appropriate control strategies and enhance the development of care-pathways to prevent visual loss from glaucoma. It is envisaged that these results will also be relevant to other countries in sub-Saharan Africa and for African-derived black populations.

## Methods

Details of all the methods used in the Nigeria Blindness Survey have been published [17] as well as data on the prevalence [7] and causes of visual impairment and blindness [8] and the prevalence and types of glaucoma in Nigeria [9].

### Study design

The sample size calculation and sampling strategy for the Nigeria Blindness Survey gave a nationally representative sample of 15,375 persons aged 40 years and above in 310 clusters across the country. The sample size was also adequate for precise estimates of glaucoma prevalence and was adequately powered for risk factor analysis for OAG.

Multi-stage sampling using probability proportional to size methods was used to select the study population. Clinical data were collected by two teams, each comprising two ophthalmologists, one optometrist and two ophthalmic nurses.

### Data collection

All participants were invited to a temporary clinic for examination. Relevant personal and demographic details and examination findings were recorded.

**Fig. 1:**
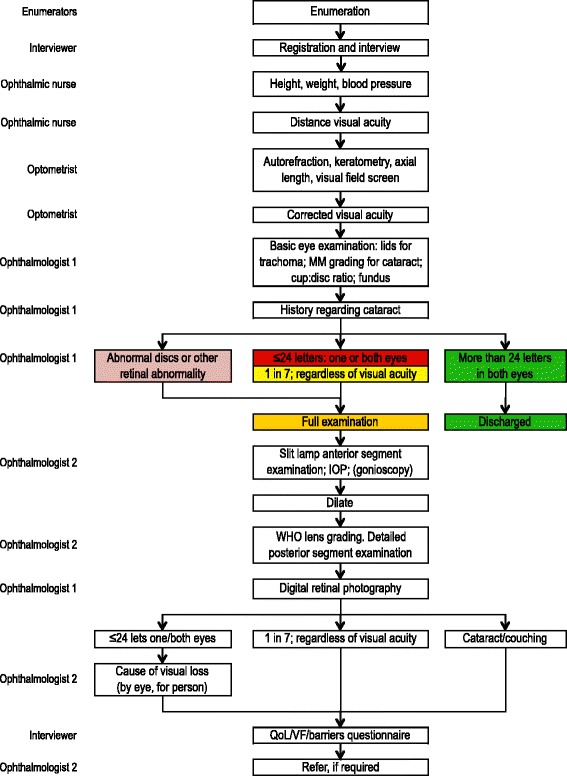
The Nigeria Blindness Survey examination flow chart

The examination flow chart (Fig. 1; adapted [17]) indicates the data collected by the team members. All participants had presenting and best-corrected visual acuity (VA) measured with a reduced logMAR tumbling E-chart, automated refraction and keratometry (Takagi ARKM-100, Takagi Seiko, Japan), frequency doubling technology (FDT) visual function testing (Carl Zeiss Meditec AG Jena Germany) and ultrasound A-scan biometry (Bioline Biometer OPTIKON 2000 S.p.A Roma, Italy). All participants had basic eye examination performed by the first ophthalmologist, and detailed ocular examination was performed by the second ophthalmologist: in those with VA of worse than 20/40 in one or both eyes; vertical cup:disc ratio (VCDR) ≥0.6 in one or both eyes or VCDR asymmetry of ≥0.2, or any retinal abnormality seen on undilated fundoscopy [17]. In addition, a subsample of 1-in-7 participants who also had the detailed examination regardless of their VA had a random blood glucose (RBG) test (OneTouch Ultra blood glucose meter, LifeScan UK).

### Risk factors assessment and classification

**Fig. 2:**
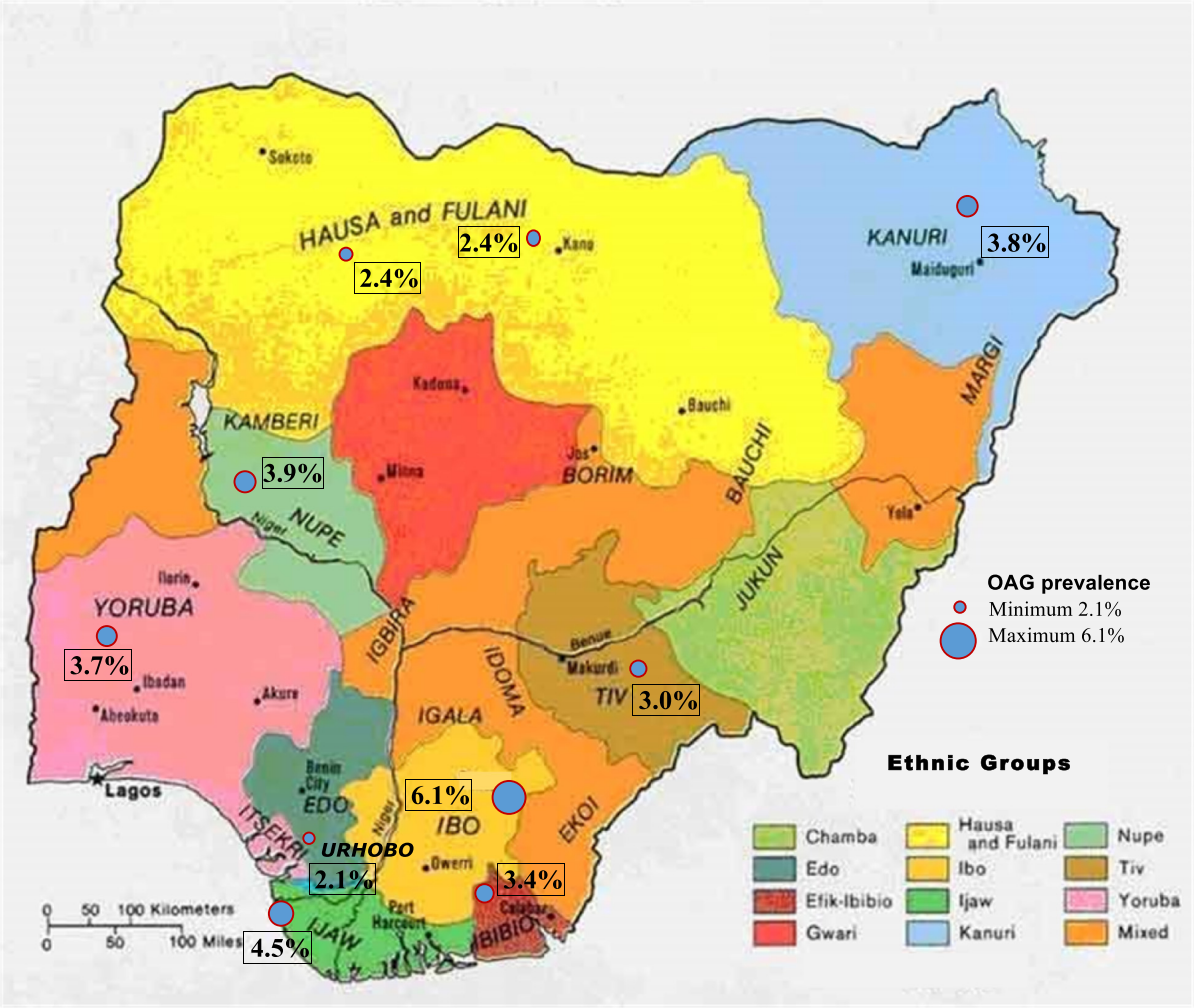
Geographical origins of ethnic groups and their open-angle glaucoma prevalence. Adapted from Map of the ethno-linguistic groups of Nigeria. Source: University of Texas Libraries; obtained from https://commons.wikimedia.org/wiki/File:Nigeria_linguistic_1979.jpg

There were five socio-demographic ‘person’ factors (age, gender, ethnic group, literacy and place of residence), six biophysical ‘person’ factors (presence of hypertension, severity of hypertension, systolic blood pressure [SBP], diastolic blood pressure [DBP], RBG and body mass index [BMI]); and three ‘ocular’ factors (axial length, IOP and mean ocular perfusion pressure [MOPP]). Age was analysed as a continuous variable and gender as a binary variable. Participants were asked about their ability to read and/or write and their ethnic group. Literacy was classified as ability to read and write or not at all and analysed as a binary variable. The geographical origins of some of the major ethnic groups are shown in Fig. 2. The Ibibio and Ijaw are from the southern Niger delta region, the Igbos and Urhobos are from the southeastern equatorial region and the Hausa, Fulani and Kanuri are from the northern savannah region. Ethnic groups with ≥200 participants (Hausa, Yoruba, Igbo, Fulani, Kanuri, Tiv, Ijaw, Urhobo, Ibibio and Nupe) were categorised and analysed separately, and the smaller ethnic groups were combined into an ‘others’ category. Urban place of residence was defined as a settlement of more than 20,000 people.

Blood pressure (BP) was recorded three times with BP Omron wrist instrument (Omron Healthcare Ltd, Milton Keynes, England) after resting for at least 10 min [18]. Average values were used for analysis. Hypertension was defined as BP ≥140/90 mmHg and severity was categorised using World Health Organization (WHO) categories: stage 1 for systolic/diastolic BP of ≥140/90 mmHg, stage 2 ≥ 160/100 mmHg and stage 3 ≥ 180/110 mmHg [19]. SBP and DBP were analysed as continuous variables. RBG was grouped as less than 11.1 mmol/L or ≥11.1 mmol/L [20]. Height was measured to the nearest tenth of a centimeter and weight was measured to the nearest 100 g using standard equipment. BMI was calculated by dividing body weight (kg) by height (m) squared and categorised according to the international classification for adults i.e., underweight (<18.5 kg/m^2^), normal (18.5–24.9 kg/m^2^), overweight (25.0–29.9 kg/m^2^) and obese (≥30.0 kg/m^2^) [21].

Axial length was measured by contact ultrasound A-scan biometry. IOP was measured using one Goldmann applanation tonometer in each of the two teams by the second ophthalmologist, using standard methods. To explore the association of vascular perfusion and OAG, the MOPP was calculated as ^2^/_3_[DBP + ^1^/_3_ (SBP-DBP)-IOP] [22]. Axial length, IOP and MOPP were analysed as continuous variables.

A person was classified as having glaucoma if one or both eyes had glaucoma. The diagnosis of glaucoma was based on the International Society for Geographical and Epidemiological Ophthalmology (ISGEO) criteria with defining values obtained from a subsample of this study population [23]: VCDR ≥0.7 or VCDR asymmetry ≥0.1 (97.5^th^ percentile) with evidence of glaucomatous visual function deficit; or VCDR ≥0.75 or VCDR asymmetry ≥0.2 (99.5^th^ percentile) when visual fields results were not available; or IOP ≥28 mmHg (99.5^th^ percentile) ± VA worse than 20/400 or known glaucoma on treatment; or if there was relative afferent pupillary defect (RAPD) associated with high IOP and/or corneal edema. The Van Herick’s anterior chamber (AC) angle estimation was performed on the slit-lamp with a narrow slit of light projected on the peripheral cornea, and was based on the relationship between the corneal slit image on the corneal surface and the AC depth. Grades 3 and 4 infer open angles and angle-closure is unlikely. The validity of the Van Herick’s method for the estimation of the AC angle to correctly identify grades 3–4 as being open angles was assessed in comparison to identification of open angles by gonioscopy. Eyes with glaucoma were classified as OAG based on open-angles seen on gonioscopy or Van Herick’s grades 3–4 in those who did not have gonioscopy.

Data for all participants classified as OAG were compared to those of the control group in analysis. Socio-demographic, ocular and biophysical factors were analysed for associations with OAG. The control group consisted of all other participants without OAG after excluding glaucoma eyes that did not have gonioscopy or Van Herick’s test findings and those with other types of glaucoma, and phthisical eyes. The algorithm for selection of OAG cases and the control group is shown in Fig. 1.

Statistical analysis was performed using Stata/IC 13.0 (Stata Corp, College Station, TX).

We examined the association between OAG and each risk factor separately and report odds ratios with 95 % confidence intervals (CI). We used logistic regression to assess the independent effect of each risk factor on OAG and report adjusted odds ratios and 95 % CI intervals. BMI was also adjusted for gender. The following variables were included in the multivariable model: age, gender, ethnic group, literacy, rural/urban residence, BP, BMI, ocular axial length, IOP and MOPP. For ocular factors, the analysis took account of within-person correlation using robust standard errors. Possible extra variation introduced by the cluster sampling strategy was also considered but it did not impact the results.

## Results

**Fig. 3:**
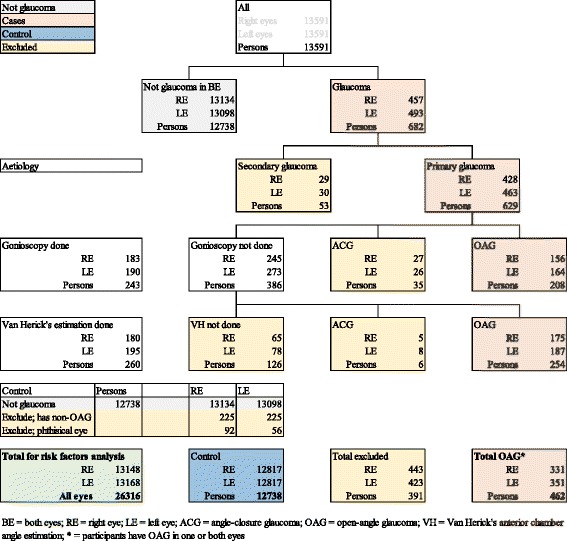
Algorithm for selection of open-angle glaucoma cases and control

A summary of completeness of data for the Nigeria Blindness Survey has been reported: for participants undergoing full examination (6397), 88 % had IOP measurement with Goldmann applanation tonometer in at least one eye [9]. In the Nigeria Blindness Survey, 950/27,182 (3.50 %) eyes of 682/13,591 (5.02 %) participants had glaucoma according to the ISGEO criteria, of which 320 eyes of 208 persons were classified as OAG by gonioscopy. 375 eyes had Van Herick’s AC angle estimation but did not undergo gonioscopy. In eyes with both values, Grades 3 and 4 Van Herick’s AC angle estimation had a 99.1 % sensitivity and 93 % positive predictive value in identifying open angles by gonioscopy. Thus, an additional 362 eyes of 254 persons were included as OAG cases as they had grades 3 or 4 Van Herick’s estimation. Hence, 462 persons (682 eyes with OAG) were included in the analysis as OAG while 12,738 persons were classified as controls (without OAG) and 391 participants were excluded (Fig. 3).

The OAG group was older and more likely to be male (Table 1). The mean age ± standard deviation (SD) of participants with OAG was significantly higher than that of controls (66.2 ± 12.3 years Vs 55.4 ± 12.1 years, *p* <0.001). Men with OAG were significantly older (mean age 67.6 years ±12.7) than women with OAG (mean age 64.8 years ±11.8; *p* = 0.02). The OAG group also had a higher proportion of participants that were of the Yoruba or Igbo ethnic group, illiterate and with hypertension and low BMI (underweight). After adjusting BMI for gender, the odds of OAG was higher in underweight women (OR 1.84, 95%CI 1.27–2.68; *p* = 0.001) but not after adjusting for age or for age and IOP. The mean ± SD IOP was higher in eyes with OAG (22 ± 11 mmHg) than in eyes without OAG (14 ± 4 mmHg, *p* <0.001). Similarly, the mean ocular axial length was longer in eyes with OAG (22.8 ± 1.09 mm) than in those without OAG (22.6 ± 0.97 mm, *p* = 0.001).

**Table 1:** Distribution of participants with and without open-angle glaucoma by socio-demographic, biophysical and ocular characteristics

	Without OAG [control]		OAG [cases]	
Total participants *N* = 12,946	*n* = 12,738 (96.5 %)		*n* = 462 (3.5 %)	
	*n*	%	*n*	%
Socio-demographic factors				
Age group (years)				
40 – 49	4760	37.4	45	9.7
50 – 59	3415	26.8	75	16.2
60 – 69	2550	20.0	124	26.9
70 – 79	1439	11.3	141	30.5
80+	574	4.5	77	16.7
Age (years) Mean ± SD	55.4 ± 12.1		66.2 ± 12.3	*p < 0.001*
Gender				
Female	6940	54.5	221	47.8
Male	5798	45.5	241	52.2
Ethnic group^a^				
Hausa	3191	25.2	78	16.9
Yoruba	2478	19.5	95	20.6
Igbo	1752	13.8	114	24.7
Fulani	801	6.3	20	4.3
Kanuri	326	2.6	13	2.8
Tiv	328	2.6	10	2.2
Ijaw	234	1.8	11	2.4
Urhobo	231	1.8	5	1.1
Ibibio	199	1.6	7	1.5
Nupe	198	1.6	8	1.8
Others	2946	23.2	100	21.7
Literacy				
Literate	5618	44.1	159	34.4
Illiterate	7120	55.9	303	65.6
Place of residence				
Rural	9883	77.6	354	76.6
Urban	2855	22.4	108	23.4
Biophysical factors				
Blood pressure (mmHg)^a^				
Normal	9343	73.8	308	67.2
Hypertension ≥140/90 mmHg	3315	26.2	150	32.8
Random blood glucose (mmol/L)^a^				
Normal	1551	97.1	98	96.1
Diabetes ≥11.1 mmol/L	47	2.9	4	3.9
Body mass index^a^				
Normal 18.5–24.9 kg/m^2^	7672	61.1	276	60.6
Underweight <18.5 kg/m^2^	1365	10.9	74	16.3
Overweight 25.0–29.9 kg/m^2^	2464	19.6	75	16.5
Obese ≥30.0 kg/m^2^	1060	8.4	30	6.6
Ocular factors^b^				
Total eyes *N* = 26,316 (100 %)	25,634 (97.4 %)		682 (2.6 %)	
Axial length (mm) Mean ± SD	22.63 ± 0.97		22.76 ± 1.09	*p = 0.001*
IOP (mmHg) Mean ± SD	14 ± 4		22 ± 11	*p < 0.001*

*IOP* intraocular pressure, *OAG* open-angle glaucoma, *SD* standard deviation

^a^missing values excluded; ^b^ocular factors distribution by eyes

In univariate analysis, increasing age was positively associated with OAG (Odds ratio [OR] 1.06, 95 % CI 1.06–1.07; *p* <0.001), as was being male (OR 1.29, 95 % CI 1.06–1.57; *p* = 0.01) (Table 2). There was 6 % higher odds of OAG with each increasing year of age. The following factors were also positively associated with OAG: Igbo and Yoruba ethnic groups, being illiterate, any hypertension and greater severity of hypertension, low BMI (underweight), longer ocular axial length, higher IOP and lower MOPP (Table 2). When adjusted for myopia, axial length remained significantly associated with OAG (OR 1.13, 95 % CI 1.02–1.25; *p* = 0.03). In multivariate logistic regression analyses, increasing age, higher IOP and Igbo ethnic group were identified as independent risk factors for OAG. The ethnic group-specific prevalence of OAG for the analysed ethnic groups are shown in Fig. 2. The Urhobo had the lowest odds of OAG (OR 0.69, 95 % CI 0.24–1.97), while the Kanuri (OR 1.81, 95 % CI 0.90–3.63; *p* = 0.10) and Igbo (OR 1.73, 95 % CI 1.18–2.56; *p* = 0.01), the highest. The Igbo ethnic group had a 73 % higher odds of OAG than the Hausa (reference group) (Table 2); and when adjusted for gender, Igbo men were 2.5 times more likely to have OAG than Hausa men (OR 2.54, 95 % CI 1,50–4.30; *p* = 0.001).

**Table 2:** Open-angle glaucoma and association with potential risk factors

	All eyes	OAG	Univariate analysis			Multivariate analysis		
	*n* (%)	*n* (%)	OR	95 % CI	*p*-value	OR	95 % CI	*p*-value
	26,316 (100 %)	682 (2.6 %)						
Socio-demographic factors								
Age (years)	(Min 40)		Reference			Reference		
Increasing age	(Max 100)		1.06	1.06–1.07	<0.001	1.04	1.03–1.05	<0.001
Gender	Female	328 (2.3)	Reference			Reference		
	Male	354 (2.9)	1.29	1.06–1.57	0.01	1.23	0.94–1.61	0.13
Ethnic group	Hausa	113 (1.7)	Reference			Reference		
	Yoruba	150 (2.9)	1.71	1.24–2.36	0.001	1.10	0.75–1.63	0.62
	Igbo	167 (4.5)	2.70	1.98–3.68	<0.001	1.73	1.18–2.56	0.01
	Fulani	31 (1.9)	1.09	0.65–1.85	0.73	1.18	0.65–2.19	0.58
	Kanuri	20 (2.9)	1.72	0.92–3.23	0.09	1.81	0.90–3.63	0.10
	Tiv	15 (2.2)	1.30	0.64–2.62	0.47	1.03	0.42–2.52	0.96
	Ijaw	14 (2.9)	1.69	0.86–3.35	0.13	1.51	0.50–4.60	0.47
	Urhobo	7 (1.5)	0.85	0.32–2.23	0.74	0.69	0.24–1.97	0.48
	Ibibio	10 (2.4)	1.43	0.62–3.27	0.40	1.29	0.58–2.89	0.53
	Nupe	9 (2.2)	1.29	0.59–2.79	0.52	1.25	0.58–2.67	0.57
	Others	144 (2.4)	1.38	1.01–1.90	0.05	1.13	0.75–1.70	0.57
Literacy	Literate	235 (2.0)	Reference			Reference		
	Illiterate	447 (3.0)	1.50	1.22–1.84	<0.001	1.06	0.79–1.42	0.70
Place of residence	Rural	527 (2.6)	Reference			Reference		
	Urban	155 (2.6)	1.02	0.81–1.28	0.88	1.14	0.85–1.54	0.38
Biophysical factors								
Hypertension	Normal	454 (2.4)	Reference			NI		
	Hypertension	223 (3.2)	1.38	1.12–1.70	0.002			
Blood pressure	Normal	454 (2.4)	Reference			Reference		
(severity of	stage 1 mild	110 (2.7)	1.15	0.88–1.51	0.31	0.87	0.57–1.33	0.52
hypertension)	stage 2 moderate	68 (3.7)	1.61	1.16–2.24	0.01	1.05	0.58–1.90	0.87
	stage 3 severe	45 (4.4)	1.91	1.27–2.88	0.002	1.05	0.45–2.45	0.90
Systolic BP	(Min 60)		Reference			NI		
	(Max 259)		1.01	1.01–1.02	<0.001			
Diastolic BP	(Min 35)		Reference			NI		
	(Max 157)		1.01	1.00–1.02	0.002			
RBG^a^	Normal	141 (4.3)	Reference			NI		
	Diabetes	4 (4.1)	0.94	0.33–2.67	0.91			
Body mass index	Normal	406 (2.6)	Reference			Reference		
(Categories)	Underweight	116 (4.0)	1.60	1.21–2.10	0.001	1.29	0.91–1.83	0.16
	Overweight	111 (2.2)	0.85	0.65–1.12	0.26	0.82	0.58–1.17	0.27
	Obese	42 (1.9)	0.75	0.50–1.12	0.16	1.18	0.71–1.96	0.52
Ocular factors								
Axial length (mm)	(Min 18.4)		Reference			Reference		
	(Max 30.0)		1.14	1.03–1.26	0.01	0.99	0.89–1.10	0.88
I OP (mmHg)	(Min 5)		Reference			Reference		
	(Max 50)		1.21	1.18–1.23	<0.001	1.22	1.18–1.25	<0.001
MOPP (mmHg)	(Min 6)		Reference			Reference		
	(Max 115)		0.96	0.95–0.97	<0.001	1.01	0.99–1.03	0.40

*BP* blood pressure, *IOP* intraocular pressure, *MOPP* mean ocular perfusion pressure, *NI* not included in multivariable models, *OAG* open-angle glaucoma, *RBG* random blood glucose

^a^tested for 1641 persons only

Systemic hypertension (BP ≥ 140/90 mmHg) was also associated with OAG, with moderate and severe hypertension having stronger and significant association with OAG in univariate analysis. After adjusting for age, IOP and other potential risk factors in a multivariable model, mildly elevated BP (stage 1) was protective of OAG compared to participants without hypertension but this was not statistically significant (OR 0.87, *p* = 0.52). There was a strong association between lower MOPP and OAG (*p* <0.001) in univariate analysis which did not persist after adjusting for age, IOP and other factors.

In univariate analysis, lower BMI was associated with 60 % greater odds of OAG (*p* = 0.001) and the odds decreased with increasing BMI. However, in the adjusted model, BMI was not statistically significant.

## Discussion

We report results of the first cross-sectional study of risk factors for OAG in sub-Saharan Africa in a large population-based, nationally representative survey in Nigeria. We did not explore the risk factors for angle-closure glaucoma, as the numbers were too few. Older age and higher IOP were independent risk factors for OAG. Additionally, an important and new finding was that the Igbo ethnicity was an independent risk factor associated with OAG, especially in men.

Significant inter-racial variation between White, Asian and Black populations has been described [11, 13, 15, 24] with the prevalence and risks of OAG being higher in Blacks. However, studies in smaller population groups in sub-Saharan Africa have not identified differences in risks of OAG by ethnic group within black populations [5, 25]. Under-powered sample sizes may be a reason why they could not detect ethnic differences in those studies. The Nigeria Blindness Survey had relatively large numbers of the main ethnic groups, giving adequate power to detect significant associations and differences within the black population. One of the potential reasons for the ethnic differences we observed may be the differential susceptibility due to larger optic discs. As reported in the normative data for the classification of glaucoma in prevalence surveys in Nigeria, the 97.5^th^ percentile VCDR for the Igbo was 0.7 compared to 0.6 for the Fulani. Interestingly, the 99.5^th^ percentile for IOP was lower for the Igbo (22 mmHg) than for the Hausa (28 mmHg) [23] and this may imply that the Igbos have thinner corneas. However, a major limitation in interpreting this difference is the absence of pachymetry to measure central corneal thickness in the Nigeria Blindness Survey, which would have enabled corrected IOP estimates for comparison. Optic disc parameters are important in OAG with respect to attenuation of structural support, axonal protection and metabolic support provided by astrocytes [26]. These quantitative parameters are heritable traits [27, 28], thus genetic variation is another plausible reason for the ethnic differential risk. Genome-wide association studies (GWAS) in the African Caribbean population of Barbados, which has a high prevalence of OAG (6.8 %, 95 % CI 6.1–7.7 % in Blacks ≥40 years old) [15], confirmed two mechanisms of gene interaction with OAG: the absence of protective genes, and the presence of predisposing alleles increased the risk for OAG [29, 30]. Although the demographics of Barbados have been dynamic, and there are other socio-demographic and lifestyle factors that influence disease incidence [31] and progression [12, 32], the historical link between the Igbos and Barbadians lends credence to the genetic basis for the ethnic differences in risk of OAG seen in Nigeria.

Another interesting observation in our study was the strong association between low BMI (underweight) and OAG, albeit only in univariate analysis: presumably because of age, as older persons have lower BMI especially when of poor socioeconomic status. Higher BMI has been reported to be protective for OAG in Barbados [12] and Rotterdam [33]. Systemic inflammatory process [34] are possible linking factors which may also result in weight loss from general debilitation.

Our study did not find significant difference in risk for OAG in urban compared to rural population as seen in urban South India where the prevalence of OAG was more than doubled than in the rural population [16]; and possible associations with hypertension or diabetes were not statistically significant.

All studies have shown increasing age to be a risk factor for OAG [12, 31, 32, 35–43]. Indeed, in the Barbados Eye Study a 4 % increase in the relative risk of OAG per year was reported [31], and comparable to 6 % higher odds of OAG per year in this study. Increasing mitochondrial dysfunction in retinal ganglion cells and increased vulnerability of the optic nerve to neurodegeneration from oxidative stress serve as possible links between ageing and increased risk for OAG [44, 45].

This study also demonstrated that higher IOP has an independent association with OAG, as in numerous other studies. Higher IOP was an independent risk factor for glaucoma despite a large number of eyes having IOPs lower than the ‘upper limit of normal’ i.e. mean (+2SD) [40]. In the National Blindness Survey, 56 % of glaucoma eyes had IOP <22 mmHg; the mean IOP in glaucoma eyes was 23 (SD12) mmHg and the mean IOP in non-glaucoma eyes was 14 (SD4) mmHg [9]. This underscores the role of IOP as a tool for monitoring response to treatment rather than as a diagnostic factor.

Men had higher odds of OAG but only in univariate analysis. An increased risk of OAG in men has been reported in previous prevalence studies in Barbados, United States [12, 32] and Singapore [43], and in a Bayesian meta-analysis, men were more likely to have POAG than women (OR 1.36, 95 % CI 1.23–1.52) [1]. Further incidence studies are needed to clarify gender differences in risks of OAG.

Some studies have addressed associations between ocular perfusion factors (IOP, BP and MOPP) and OAG which suggest that vascular insufficiency is an important factor in OAG [31, 38, 46], as was in our study, higher BP and lower MOPP were significantly associated with higher odds of OAG.

Longer ocular axial length has been associated with OAG [37, 47]. In the Nigeria Blindness Survey axial length was longer in OAG eyes and was significantly associated with OAG, but this was not an independent risk factor after adjusting for age, IOP and other variables. In our study we assessed axial length rather than myopia as a potential risk factor because there was a high prevalence of nuclear lens opacities (8.8 %, 95 % CI 7.5–10.1) [48] which would increase the risk of index myopia; and a relatively low prevalence of myopia ≤0.5D (after excluding persons with lens opacity, 9.4 %, 95 % CI 8.7–10.2) [49].

A strength of the Nigeria Blindness Survey is that it was nationally representative and had a large sample size with adequate power to detect statistical associations. A range of ethnic groups was represented in large enough numbers to allow comparison of risk between the largest ethnic groups in Nigeria. As part of the study protocol, not all participants had gonioscopy done and we did not record the presence of pseudoexfoliation (PXE). Hence, PXE was not assessed as a risk factor for OAG. In addition, some eligible participants did not have gonioscopy performed due to damage to the mirrors on the gonioscopy lenses by high humidity; and did not have Van Herick’s AC angle estimation due to structural ocular pathology. Another limitation was that IOP was measured once and it was not interpreted using central corneal thickness, which was not measured. Additionally, visual field analysis was by FDT and participants classified as glaucoma did not undergo Humphrey visual field analysis (HFA). We were also not able to obtain information on duration of hypertension, history of cardiovascular disease or use of antihypertensive medication. However, this may not have a significant impact as only 14 % of participants reported being hypertensive [18]. Additionally, we did not obtain information on family history of glaucoma which would not have been reliable in this context. Indeed, only 5.6 % of those identified with OAG knew they had the condition [9].

This is the first time that an association of OAG has been observed with some ethnic groups. It is imperative that this finding be replicated in further studies as it may be a chance finding. While cultural or other practices might underlie the differences, or failure to fully adjust for confounders, given the relative lack of environmental factors identified to date for OAG, these observations suggest the need for a molecular genetics study of glaucoma in Nigeria. This might be included within a follow-up study on the cohort of the Nigeria Blindness Survey to explore the natural history and incidence of glaucoma, and the influence of immunological markers of inflammation.

## Conclusion

This study gives us risk factors data on OAG and confirms that OAG is a public health problem in people ≥40 years. As a public health strategy, opportunistic eye examination, case detection and examination for OAG need to be performed on all people aged ≥40 years and the ethnic groups most at risk.

## Abbreviations

AC, Anterior chamber; BMI, Body mass index; BP, Blood pressure; CI, Confidence intervals; DBP, Diastolic blood pressure; FDT, Frequency doubling technology; GWAS, Genome-wide association studies; IOP, Intra-ocular pressure; ISGEO, International Society of Geographical and Epidemiological Ophthalmology; MOPP, Mean ocular perfusion pressure; OAG, Open-angle glaucoma; OR, Odds ratio; RAPD, Relative afferent pupillary defect; RBG, Random blood sugar; SBP, Systolic blood pressure; SD, Standard deviation; VA, Visual acuity; VCDR, Vertical cup:disc ratio; WHO, World Health Organization.
